# Editorial: Epilepsy and Neurodevelopmental Diseases

**DOI:** 10.3389/fncel.2020.00255

**Published:** 2020-09-25

**Authors:** Gabriele Ruffolo, Erwin A. Van Vliet, Eleonora Aronica, Eleonora Palma

**Affiliations:** ^1^Department of Physiology and Pharmacology, Istituto Pasteur-Fondazione Cenci Bolognetti, University of Rome Sapienza, Rome, Italy; ^2^Istituto di Ricovero e Cura a Carattere Scientifico San Raffaele, Cassino, Italy; ^3^Center for Neuroscience, Swammerdam Institute for Life Sciences, University of Amsterdam, Amsterdam, Netherlands; ^4^Department of (Neuro)Pathology, Amsterdam UMC, University of Amsterdam, Amsterdam, Netherlands; ^5^Stichting Epilepsie Instellingen Nederland, Heemstede, Netherlands

**Keywords:** GABA, ion channels, brain immaturity, synaptic transmission, neurodevelopment, epilepsy

The association between epilepsy and neurodevelopmental diseases is well-recognized and has gained significant attention in the field of neuroscience in recent years. One of the main reasons for this interest is the need for a better understanding of the events that lead to the development and maturation of the CNS. This is a fundamental and necessary basis for potential breakthrough strategies that could guide novel and more effective disease-modifying therapeutic approaches to neurodevelopmental syndromes that are frequently characterized by severe and drug-resistant epilepsy.

The perspective of such new therapeutic strategies is very promising. At the *state-of-the-art*, patients afflicted by these rare neurodevelopmental disorders mostly rely on “symptomatic” approaches that mitigate seizures and other major symptoms but do not target the underlying biological causes of the disease.

The study of this vast field of research is extremely complex and requires a multidisciplinary approach, from neuropathological to molecular and functional studies since even “simple” triggering events (e.g., a genetic mutation) during critical periods of brain development can lead to widespread effects on brain morphological and functional features.

This issue represents the concept of an integrated, interdisciplinary translational approach to epileptic syndromes of neurodevelopment. For instance, Rosch et al. thoroughly describe invertebrate animal models such as zebrafish and *drosophila* which can be used as tools to investigate the early stages of neurodevelopment. Even though these “simple” models are far from the complexity of the human brain, they offer superior manageability to mammalian models, allowing researchers to perform single-cell resolution studies on whole-brain imaging, thus tracking the effects of a pathogenic event from the “larval” stages to the mature brain.

Since the mechanisms by which an insult determines pathological events during neurodevelopment are not always univocal, they can be explored from different angles, using different methods and multidisciplinary perspectives in research design. For example, considering their pivotal role in neurotransmission, it is not surprising that many researchers have made efforts to characterize ion channels. Here, two original research articles by Levinson et al. and by Mao et al. followed this path.

The first by Levinson et al. supports the role of metabotropic GABA_B_ receptors (GABA_B_Rs) in pediatric focal cortical dysplasia (FCD) and tuberous sclerosis complex (TSC) through an electrophysiological study of patients' brain slices. This research strengthened the hypothesis that there is significant participation of GABA_B_Rs in synaptic inhibition in the aforementioned pathologies, highlighting the importance of studying possible therapeutic targets in a “comparative” fashion, since the permissive role of GABA_B_Rs was observed in FCD and TSC, but not in non-dysplastic tissues.

The second study by Mao et al. reported two novel mutations in the *KCTN2* gene, encoding for K_Na_1.2 subunit of the sodium-dependent voltage gated potassium channel K_Na_. This protein frequently mutates in Epilepsy of Infancy with Migrating Focal Seizures (EIMFS) and this study deepened the knowledge of disease genetics and physiology, thus facilitating future studies of the mutated channel in cellular models.

The review by van Loo and Becker describes acquired and genetic channelopathies from an original point of view. This review makes clear that the direct malfunction of a channel protein is not the only problem, as many events precede the incorporation of the channel in the cellular membrane, such as regulation of mRNA transcription, micro-RNA interference, and epigenetic mechanisms such as DNA methylation.

In addition, another review by Fattorini et al. tackles the other relevant mechanisms that can modulate neurotransmission: neurotransmitter reuptake. The authors have reviewed recent findings on the localization of GAT-1 to underline the widespread distribution of the transporter in the CNS as an alternative to traditional ideas about its neuron-specific expression. This is an important contribution to a field that is continuously evolving.

The research article by Duy et al. proposed an interesting approach, aiming to restore an optimal function of KCC2 to enhance neuronal chloride extrusion, making neurons less prone to generate epileptic discharges. Such research also raises the question of whether similar strategies may prevent epileptogenesis and cognitive impairment when applied early during development.

The pathologic traits in the cytoarchitecture of the diseased brain are also explored in this issue. Liu et al. performed a proteomics study that described how the expression of Rho GTPases in hippocampal dentate granule cells may support the abnormal migratory activity observed in mesial temporal lobe epilepsy, determining granule cell dispersion. These dysmaturative processes reproduce events, characterizing early developmental stages, with detrimental consequences. These findings clearly show that the dysmaturation is not confined to the early stages of development but it is also a feature of adult mesial temporal lobe epilepsy. Liu et al. explored the impact of an early developmental dysregulation of the mechanistic target of rapamycin (mTOR) pathway, as a result of mutations of *STRADA* (pseudokinase STE20-related kinase adaptor alpha, an mTOR regulator), leading to a continuous overactivation of mTOR signaling. In particular, they studied the malfunction of S*TRADA* with complementary techniques such as CRISPR-edited *Strada* mouse N2a cells and induced pluripotent stem cells (iPSCs). This study highlighted the novel contribution of this protein to pathologic hallmarks of mTORopathies such as increased cell size, neuronal hyperexcitability, and impaired cortical lamination.

The list of promising targets that require further investigation is much longer, as indicated in papers by Allocco et al. and Fassio et al.. The first research article defined a role for the impairment of Na^+^/K^+^ ATPase function in the pathogenesis of congenital hydrocephalus trough whole-exome sequencing, bioinformatics, and computational modeling. Indeed, these authors, by characterizing novel mutations of α3 subunit of Na^+^/K^+^ ATPase, have described, for the first time, a link between this protein and congenital hydrocephalus.

In their review, Fassio et al. highlighted the role of autophagy in neurodevelopment and epilepsy as a key process involved in neurogenesis, neuronal polarity, and synaptic function. Notably, these authors shed new light on this physiological mechanism, suggesting that defective autophagy may represent an additional therapeutic target for epileptic neurodevelopmental diseases.

In conclusion, this Research Topic combines different fields of neuroscience that analyze the correlation between epilepsy and neurodevelopment from different points of view. Therefore, as well as making significant contributions to research, this topic highlights the need for an integrated approach—from anatomy and pathology to molecular biology and electrophysiology—to speed-up the progress in this research field.

## Author Contributions

All authors listed have made a substantial, direct and intellectual contribution to the work, and approved it for publication.

## Conflict of Interest

The authors declare the absence of any commercial or financial relationship that could be construed as a potential conflict of interest.

